# Possible mechanism of inotropic and chronotropic effects of *Rosa damascena* on isolated guinea pig heart

**DOI:** 10.1186/2008-2231-21-38

**Published:** 2013-05-20

**Authors:** Mohammad Hossein Boskabady, Alaleh Vatanprast, Haydar Parsaee, Morteza Boskabady

**Affiliations:** 1 Applied Physiology Research Centre, School of Medicine, Mashhad University of Medical Sciences, Mashhad, 9177948564, Iran; 2 Department of Physiology, School of Medicine, Mashhad University of Medical Sciences, Mashhad, 9177948564, Iran; 3 Pharmacological Research Centre of Medicinal Plants, School of Medicine, Mashhad University of Medical Sciences, Mashhad, Iran; 4 Department of Pharmacology, School of Medicine, Mashhad University of Medical Sciences, Mashhad, Iran; 5 Department of Physiology, School of Medicine, Mashhad 91735, IRAN

**Keywords:** *Rosa damascena*, Inotropic effect, Chornotropic effect, β-adrenoceptor, Isolated heart

## Abstract

**Background:**

The possible mechanism(s) of inotropic and chronotropic effects of the extract from *Rosa damascena* (*R. damascena*) on heart was examined*.*

**Methods:**

Inotropic and chronotropic effects of four concentrations of the extract from *R. damascena* and isoprenaline were examined in isolated guinea-pig hearts perfused through aorta in a Langendorff model*.* All measurements were performed in three different groups: 1) In the presence and absence of propranolol, 2) In the presence and absence of methacholine and 3) In the presence of diltiazem (n = 12 for each group).

**Results:**

In all groups both isoprenaline and the extract caused an increase in heart rate and contractility (p < 0.05 to p < 0.001). Only in group 1, the final concentration of isoprenaline in the absence of propranolol caused significant greater increase in heart rate compared to the extract (207.6 ± 11.0 compared to 162.6 ± 11.8, p < 0.01). The percent increase in heart contractility due to the final concentration of the extract in the absence (362.4 ± 36.9 compared to 227.7 ± 31.6, p < 0.01) and presence of propranolol (577.1 ± 62.9 compared to 357.5 ± 45.6, p < 0.001) in group 1 and absence (403.7 ± 42.1 compared to 244.8 ± 18.9, p < 0.005) and presence of methcholine (499.88 ± 64.64 compared to 323.90 ± 44.49, p < 0.05) in groups 2 was significantly greater than the increase caused by isoprenaline.

**Conclusions:**

The results of this study suggest that inotropic and chornotropic effect of *R. damascena* is possibly due to the stimulatory effect of this plant on beta-adrenoceptors.

## Background

Heart failure is a major cardiovascular problem, a syndrome with various etiologies that results in impaired quality of life and shortened life expectancy [1]. Positive inotropic drugs have therapeutic value in heart failure [2] but currently available classes of cardiotonic drugs limit their clinical usefulness [3]. Major available inotropic drugs include sympathetic stimulants, phosphodiesterase (PDE) inhibitors, digitalis glycosides and calcium channels openers [1].

Heart rate reflects the dynamic balance between sympathetic and parasympathetic divisions of autonomic nervous system. Sympathetic stimulators or parasympathetic inhibitory agents are able to increase heat rate. Chronotropic incompetence (CI) is the inability of heart to increase its rate commensurate with increased activity or demand and is common in patients with cardiovascular disease with particular emphasis on its prominent role in HF. Therefore positive chrontropic agents could be of therapeutic values in these patients [4].

*R. damascena* is cultivated in all over the world including Iran (especialy in Kashan) for visual beauty and its scent [5]. Flowers of this plant are large, showy and colorful. This plant contains carboxylic acid [6], terpene, myrcene, and vitamin C [7]. Kaemfrol and glycoside are two other constituents of this plant [8]. The plant also containe phenolic compound which could be used as anti-oxidant [8].

Therapeutic effects such as treatment of abdominal and chest pain, strengthening heart [9], treatment of menstrual bleeding, digestive problems [10], and anti inflammation has been described for *R. damascena*. A decoction of the root of *R. damascena* as a cough remedy to ease children’s cough [5] and the plant as a gentle laxative are also used [11]. The anti HIV [12], hypnotic [13,14], antispasmodic [15], anti-inflammatory, analgesic [16], antioxidant, hepatoprotective, antidiabetic [17,18] and antidepressant [19] effects for this plant were also reported. The antitussive [20] and relaxant effect of *R. damascena*[21] and its fractions [22] on guinea pig trachea were also demonstrated. In a recent review article, different pharmacological effect of *R. damascena* was summerized [23]. Cardiotonic effect was previously described for *R. damascena*[24]. In a recent study, the effect of the plant on heart rate and contractility was demonstrated [25].

In the present study, the possible mechanism(s) of ionotropic and chronotropic effects of the aqueous-ethanolic extract from *R. damascena* was examined.

## Methods

### Plant and extracts

*R. damascena* was the same plant used in our previous study [25]. A voucher specimen was preserved in the Herbarium of the school of Pharmacy, Mashhad University of Medical Sciences (Herbarium No: 254-1804-01). The Aqueous-ethanolic extract was prepared as previously described for *R. damascena* and other plants [21,25,26]. The plant ingredient concentration in the final extract was 10 g%.

### Preparation of the isolated hearts

Dunkin Hartley guinea pigs of either sex, with a body weight of 400 - 500 g, were used in the present study (Razi Institute, Mashhad, Iran). Preparation of isolated heart was carried out exactly as previously described [25,27,28]. The hearts were perfused with K-H buffer solution (37°C, pH 7.4, saturated with 95% O2 and 5% CO2) through aorta on a modified Langendorff apparatus at a constant perfusion pressure of 70 mmHg [25]. The K-H buffer solution contained the following ingredients (in mMol/L): NaCl 118, NaHCO3 25.0, KCl 4.7, KH2PO4 1.2, MgSO4 1.2, CaCl2 2.5, and glucose 11.0 (Merck, Germany) and equilibrated with 95% O2 + 5% CO2 at 37°C. All the hearts were first perfused with K-H solution for 20-30 min for stabilization in a Langendorff apparatus and then the effects of extract from *R. damascena* and also isoprenaline were studied.

### Protocol of experiments

Heart rate and heart contractility were measured in the presence of four different concentrations of aqueous-ethanolic extract from *R. damascena* (0.1, 0.2, 0.4 and 1.0 mg% from the extract), isoprenaline sulphate (Sigma Chemical Co. Ltd UK), (1, 10 nM, 0.1 and 1 μM) and compared to baseline values. Each concentration of the solutions was given as one-minute intracoronary infusion and its inotropic and chornotropic effects were recorded in last 30 sec, similar to previous studies [25,27]. For infusion of each concentration of the extract or isoprenaline, Krebs solution containing that concentration was infused instead of Krebs solution alone. Both heart rate and contractility in the absence of pharmacological intervention were reproducible which were served as its own control [25,27]. The effects of different solutions were tested with three different experimental designs (n = 12 for each group) as follows: In the presence and absence of 1 μM propranolol hydrochloride (Sigma Chemical Co. Ltd UK) (group 1), in the presence and absence of 1 μM methacholine hydrochloride (Sigma Chemical Co. Ltd UK) (group 2), and in the presence of 10 μM diltiazem (group 3).

Three different series of animal hearts were used for examination of three groups of experiments. In each heart, the effects of the aqueous-ethanolic extract and isoprenaline were evaluated randomly with a 30-min resting period while the heart is perfused with Krebs solution. The heart rate (HR) and contractility were recorded on a kymograph (ET8 G-Boulitt, Paris) and measured after fixation similar to previous studies [25,27]. The local Animal Research Committee of Mashhad University of Medical Sciences approved the experimental procedures used in the present study. Animal care procedures were performed in accordance with the National Institutes of Health Guide for Care and Use of Laboratory Animals.

### Statistical analysis

The data were expressed as mean ± SEM. The data of each concentration of the extract in each group were compared with those of isoprenaline using paired "t" test. The percent increase in heart rate and contractility due to final concentration of the extract and isoprenaline in each group were also compared using paired "t" test. The data obtained in the presence of different concentrations and baselines were compared using ANOVA test in each group. The effect of the aqueous extract and isoprenaline were related to the concentrations of the solutions using least square regression. Significance was accepted at p < 0.05.

## Results

### The effect of the extract and isoprenaline on heart rate

**Figure 1:**
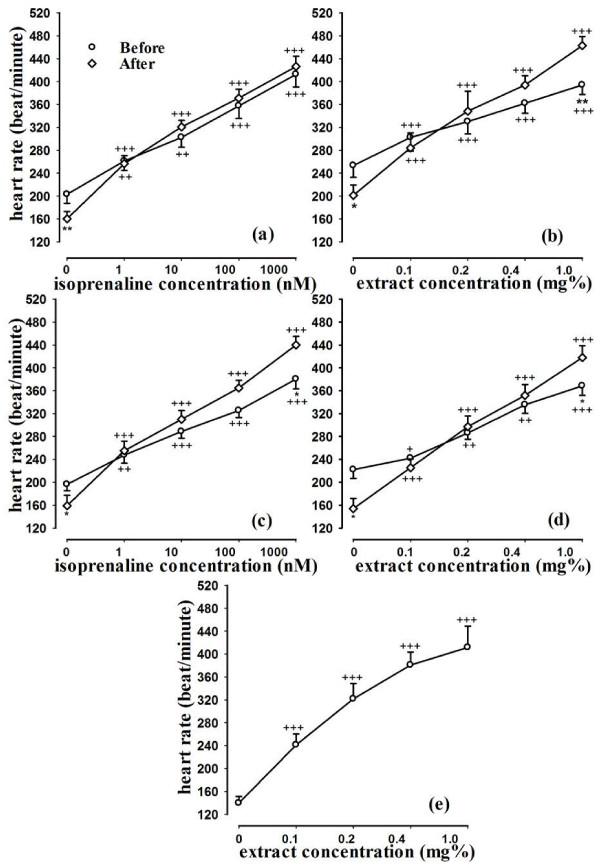
**Concentration response curves of aqueous-ethanolic extract from *R. damascena *and isoprenaline, on heart rate of guinea pigs in group 1 (in the presence and absence of propranolol), (a for the isoprenaline and b for the extract), group 2 experiments (in the presence and absence of methacholine), (c for the isoprenaline and d for the extract) and group 3 (in the presence diltiazem), (e for the extract).** Statistical differences between the data in the presence and absence of propranolol and methacholie; *; p < 0.05, **; p < 0.01. Statistical differences between the data of each concentration of extract or isoprenaline compared to baseline value; +; p < 0.05, ++; p < 0.01, +++; p < 0.001. (n = 12 for each group).

In experimental group 1, both isoprenaline and the extract caused concentration dependent and significant increase in heart rate (p < 0.001 for all cases). In addition, all concentrations of isoprenaline and the extract significantly reversed the effect of propranolol on heart rate (p < 0.001 for all concentrations), (Figure 1). In experimental group 2, also isoprenaline and the extract caused concentration dependent increase in heart rate (p < 0.001 for all concentrations). All concentrations of isoprenaline and the extract significantly reversed the effect of methacholine on heart rate (p < 0.001 for all concentrations), (Figure 1). In experimental group 3, all concentrations of the extract significantly reversed the effect of diltiazem on heart rate (p < 0.001 for all concentrations), (Figure 1). In group 1, the percent increase in heart rate due to the final concentration of isoprenaline (1 μM) in the absence of propranolol was significantly greater than that of the extract (p < 0.01), (Table 1). In group 2, there was no significant difference between the percent increase in heart rate caused by the final concentration of isoprenaline and extract both in absence and presence of methacholine (Table 1).

**Table 1:** Increased heart rate and contractility due to the final concentration pof the extract and isoprenaline (precent propertion to baseline values in group 1 and 2 experiments and the statistical differences between the extract and isoprenaline)

**Experimental design**		**Isoprenaline**	**Extract**	**St.Dif**	**Isoprenaline + An.**	**Extract + An.**	**St.Dif**
Group 1	Rate	207.6 ± 11.0	162.6 ± 11.8	p < 0.01	281.8 ± 21.9	248.6 ± 21.0	NS
	Contractility	227.7 ± 31.6	362.4 ± 36.9	p < 0.01	357.5 ± 45.6	577.1 ± 62.9	p < 0.01
Group 2	Rate	192.8 ± 12.9	204.2 ± 10.2	NS	308.7 ± 31.0	303.4 ± 36.6	NS
	Contractility	244.8 ± 18.9	403.7 ± 42.1	p < 0.005	323.9 ± 44.5	499.9 ± 64.6	p < 0.05

Data were expressed as mean ± SEM. An.: antagonist. St.Di: Statistical difference.

### The effect of the extract and isoprenaline on heart contractility

**Figure 2:**
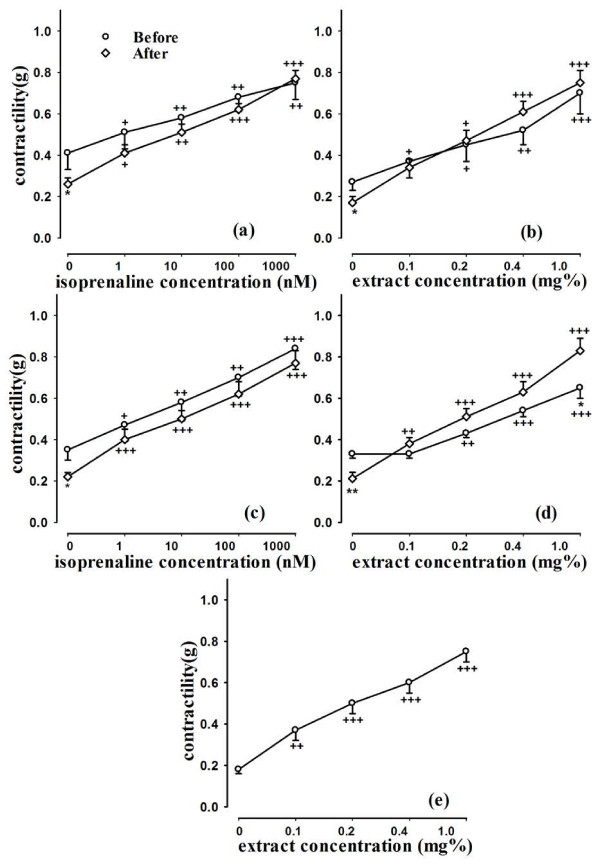
**Concentration response curves of aqueous-ethanolic extract from *R. damascena *and isoprenaline, on heart contractility of guinea pigs in group 1 (in the presence and absence of propranolol), (a for the isoprenaline and b for the extract), group 2 experiments (in the presence and absence of methacholine), (c for the isoprenaline and d for the extract) and group 3 (in the presence diltiazem), (e for the extract).** Statistical differences between the the data in the presence and absence of propranolol and methacholie; NS: non-significant difference, *; p < 0.05, **; p < 0.01. Statistical differences between the data of each concentration of extract or isoprenaline compared to baseline value; +; p < 0.05, ++; p < 0.01, +++; p < 0.001. (n = 12 for each group).

In experimental group 1, both isoprenaline and the extract caused concentration dependent and significant increase in heart contractility (p < 0.05 to p < 0.001). All concentrations of isoprenaline and the extract significantly reversed the effect of propranolol on heart contractility (p < 0.05 to p < 0.001), (Figure 2). In experimental group 2 also isoprenaline and the extract caused concentration dependent increase in heart contractility (p < 0.05 to p < 0.001). All concentrations of isoprenaline and the extract significantly reversed the effect of methacholine on heart contractility (p < 0.005 to p < 0.001), (Figure 2). In experimental group 3, all concentrations of the extract significantly reversed the effect of diltiazem on heart contractility (p < 0.05 to p < 0.001), (Figure 2). The percent increase in heart contractility due to the final concentration of the extract (1.0 mg%) in the absence and presence of propranolol was significantly greater than that of isoprenaline (p < 0.01 for both cases) in group 1 (Table 1). The percent increase in heart contractility due to the final concentration of the extract in the absence and presence of methacholine was also significantly greater than that of isoprenaline (p < 0.05 and p < 0.001 for the absence and presence of methacholine), (Table 1).

### Differences in the heart rate and contractility before and after propranolol, methacoline and diltiazem

Propranolol, methacholine and diltiazem caused significant reduction in heart rate (p < 0.05 for propranolol and methacholine and p < 0.005 for diltiazem) and its contractility (p < 0.05 for all cases). However, the increase in heart rate and contractility due to the final concentration of isopranaline and the extract in the presence of propranolol and methacholine was greater than the increase in absence of them in most cases which were significant in some cases (p < 0.01 for heart rate due to the extract after propranolol, p < 0.05 for heart rate due to isoprenaline and the extract after methacholine and p < 0.05 for heart contractility due to the extract after methacholine), (Figures 1 and 2).

### Relationship between concentration and the effect of the aqueous-ethanolic extract and diltiazem

Significant correlations were seen between both heart rate and heart contractility and concentration of isoprenaline and the extract in all three groups of experiments (P < 0.01 to p < 0.001), (Table 2).

**Table 2:** **Correlation between the effects of aqueous-ethanolic extract from *R. damascene *and isoprenaline on heart rate and contractility of isolated guinea pig heart with concentrations in three groups of experiments**

**Groups**		**Extract**		**Extract + An.: antagonist.**		**Isoprenaline**		**Isoprenaline + An.: antagonist**	
		**R**	**P value**	**R**	**P value**	**r**	**P value**	**r**	**p value**
Group 1	HR	0.4	p < 0.005	0.6	p < 0.001	0.7	p < 0.001	0.7	p < 0.001
	Cont	0.4	p < 0.01	0.6	p < 0.001	0.3	p < 0.05	0.7	p < 0.001
Group 2	HR	0.6	p < 0.001	0.6	p < 0.001	0.7	p < 0.001	0.7	p < 0.001
	Cont	0.7	p < 0.001	0.7	p < 0.001	0.3	p < 0.05	0.6	p < 0.001
Group 3	HR			0.5	p < 0.001				
	Cont			0.5	p < 0.001				

For abbreviations see Table 1.

## Discussion

Concentration dependent increase in both heart rate and heart contractility due to the aqueous-ethanolic extract of *R. damascena* was observed in the present study with prominent effect of the extract on heart contractility (ionotropic effect).

The effect of the extract was also examined on pre-treated heart with propranolol, methacholine and diltiazem to explore the possible mechanism(s) for inotropic and chornotropic effects of the plant. The effect of propranolol on both heart rate and contractility was significantly and in a dose dependent manner reversed by the extract which was more prominent on heart contractility. In fact, propranolol led to a significant reduction in heat rate and contractility. Even low concentration of isoprenalie and the extract caused less increase in heat rate and contractility in presence of propranolol compared to its absence. However, inceasing the concentration of isoprenaline and the extract overcome the effect of propranolol simillary which is a well known characteristic of competitive antagonist and agonist interaction. In pre-treated heart with propranolol, the effect of the extract on heart rate was lower but its effect on contractility was greater than that of isoprenaline. In experimental group 2, the extract, significantly and in a dose dependent manner, reversed the effect of methacholine on both heart rate and contractility with a greater effect on heart contractility rather than heart rate. The effect of the extract on contractility of pre-treated heart with methacoline was also greater than that of isoprenaline. In experimental group 3 also the extract, significantly and in a dose dependent manner, reversed the effect of diltiazem on both heart rate and contractility. In this group the effect of the extract on contractility of pre-treated heart with diltiazem was also greater than the effect on heart rate.

The results of the present study increased heart rate and contractility as well as significant correlation between concentrations of the extract and its effects on heart rate and heart contractility and its similar effect to isoprenaline support the choronotropic and inotropic effect of hydro-ethanolic extract of *R. damascena* found in our previous study [25]. The results also showed that the extract reversed the effects of pre-treated heart with propranolol, methacholine and diltiazem. These findings suggest that the possible mechanisms of action of the extract from *R. damascena* on heart are β-adrenoceptor stimulatory, calcium channel opening activity or inhibitory effect on cholinergic receptors [29]. The findings on pre-treated heart with propranolol were also supported by our previous study [25]. However, if the hydro-ethanolic extract has a blockade effect on cholinergic receptors, it should increase heart rate more than contractility. The results of the present study showed that the extract affects heart contractility more than heart rate. Therefore the inhibitory effect of the extract on cholinergic receptors of the heart is excluded. Although the extract reversed the effect of diltiazem on both heart rate and contractility which may indicate an opening effect of the extract on calcium channels. With regard to the greater effect of calcium channel opener drugs on heart contractility than heart rate which is similar to the results of the present study, the extract of *R. damascena* may have an opening effect on calcium channels of heart.

However, the most possible mechanism of action of hydro-ethanolic extract on heart is suggested to be the stimulatory effect on β-adrenoceptor because the extract increased both heart rate and contractility. In addition, if the extract has a stimulatory effect on β-adrenoceptors, it can reverse the effect of both methacholine and diltiazem in a functional antagonism manner. However, the inhibitory effect of the extract on cholinergic receptors and its opening effect on calcium channels could not be fully excluded because the hydro-ethanolic extract is composed of several constituents and could have several mechanisms of action. In fact a muscarinic receptor inhibitory effect for *R. damascena* was shown in a recent study [15]. Therefore, the inhibitory effect of the plant on muscarinic receptors and its opening effect on calcium channels should be investigated in more detailed studies. In addition, the effect of different fractions of the extract also needs to be studied on heart activities and the mechanism(s) of their actions should be explored in further studies.

The other possible mechanism of action of the plant is an increased cAMP level like phosphodiestrase III inhibition [30] or forskolin-like action [31] of *R. damascena*. These possible mechanisms of action of the plant also should be examined in further studies. Because of the solution used for heart perfusion, contained ca2+ ion it is possible that the extract or drug could interact with calcium ion and calcium chelating is formed. However if such interactions had happened, the decrease in heart rate and specially contractility should have been observed. The results showed heart rate and contractility increasing effects for isoprenaline and the extract which suggest the abscence of interactions between the extract or drug and calcium ion.

Choronotropic and inotropic effect of the extract of *R. damascena*observed in the present study with a possible stimulatory effect on β-adrenoceptors may represent a pharmacological action and a therapeutic value in various cases of cardiac impairment such as lack of activator of calcium (e.g: hypocalcemia), [32,33].

## Conclusions

In conclusion, this study supports a potent inotropic and chornotropic effect for *R. damascena* on isolated guinea pig heart with possible stimulatory effect on β-adrenoceptor of isolated guinea pig heart. In addition the results may also suggest an opening effect on calcium channels and/or an inhibitory property for the plant on muscarinic reptors of isolated heart.

## Competing interest

The authors declare that they have no competing interest.

## Authors’ contributions

BMH: study design, supervision of experiments, help in statistical analysis, preparation of manuscript. VA: performance of experiment, help in statistical analysis and manuscript preparation. PH: help in study design and supervision of experiments. BM: help in statistical analysis, preparation of Tables and Figures. All authors read and approved the final manuscript.
